# The COVID-19 Pandemic as a Lesson: WHO Actions Versus the Expectations of Medical Staff—Evidence from Poland

**DOI:** 10.3390/jcm15030988

**Published:** 2026-01-26

**Authors:** Sławomir Lewicki, Justyna Bień-Kalinowska, Michał Zwoliński, Aneta Lewicka, Łukasz Szymański, Julia Weronika Łuczak, Natasza Blek, Piotr Świtaj

**Affiliations:** 1 Institute of Outcomes Research, Maria Sklodowska-Curie Medical Academy in Warsaw, Pl. Żelaznej Bramy 10, 00-136 Warsaw, Poland; justyna.bien-kalinowska@uczelniamedyczna.com.pl (J.B.-K.); michal.zwolinski@uczelniamedyczna.com.pl (M.Z.); 2 Military Centre of Preventive Medicine Modlin, 05-100 Nowy Dwór Mazowiecki, Poland; 3 Department of Molecular Biology, Institute of Genetics and Animal Biotechnology, Polish Academy of Sciences, 05-552 Magdalenka, Poland; l.szymanski@igbzpan.pl (Ł.S.); j.luczak@igbzpan.pl (J.W.Ł.); 4 Department of Nanobiotechnology, Institute of Biology, Warsaw University of Life Sciences, Ciszewskiego 8, Bldg. 23, 02-786 Warsaw, Poland; 5 Institute of Clinical Sciences, Maria Sklodowska-Curie Medical Academy in Warsaw, Pl. Żelaznej Bramy 10, 00-136 Warsaw, Poland; natasza.blek@uczelniamedyczna.com.pl (N.B.); piotr.switaj@uczelniamedyczna.com.pl (P.Ś.)

**Keywords:** COVID-19 pandemic, World Health Organization (WHO) guidelines, personal protective equipment (PPE) implementation, healthcare workforce safety, pandemic response and preparedness

## Abstract

**Background/Objectives**: The COVID-19 pandemic exposed global weaknesses in healthcare preparedness and highlighted the pivotal role of the World Health Organization (WHO) in coordinating responses and issuing technical guidance. Among these, the document “Rational use of personal protective equipment (PPE) for COVID-19 and considerations during severe shortages” (December 2020) aimed to standardize PPE use amid global scarcity. This study assessed the awareness, implementation, and perceived usefulness of this WHO guidance among Polish healthcare personnel and evaluated discrepancies between the WHO expectations and workplace realities. **Methods**: A cross-sectional, anonymous online survey was conducted between July and September 2025 among employees of 243 randomly selected healthcare facilities in Poland (constituting 20% of all hospitals). The original 24-item questionnaire covered the demographics, awareness and implementation of the WHO PPE guidelines, and perceptions of their effectiveness during and after the pandemic. Data were analyzed descriptively. **Results**: A total of 542 healthcare workers participated, predominantly nurses (56.8%) and physicians (12.2%), with 86.8% being female and 59.3% having over 20 years of experience. Most respondents (76.5%) reported familiarity with the WHO PPE document, and 63.1% confirmed its implementation in their institutions. Over two-thirds (68.0%) reported that the guidelines improved their sense of safety at work. The main barriers to implementation included staff shortages (52.9%) and insufficient local guidance (20.6%). In 2025, 52.3% continue to apply the WHO recommendations, and 70.8% believe they remain relevant in current practice. However, 80.2% indicated that the WHO guidance should be more closely adapted to local conditions. **Conclusions**: The WHO PPE guidance was widely recognized and reported as implemented by respondents from participating healthcare facilities, contributing to improved preparedness. Nonetheless, limited institutional support and inadequate local adaptation reduced implementation effectiveness. Future WHO recommendations should better align with national healthcare contexts to enhance preparedness for future crises.

## 1. Introduction

The World Health Organization (WHO), established in 1948 as the first specialized agency of the United Nations, plays a central role in global health governance [1]. Its core areas of work include health systems, life-course health, communicable and noncommunicable diseases, emergency preparedness, surveillance and response, and corporate services. By setting and monitoring international health standards and coordinating diverse actors, the WHO supports governments in strengthening health governance. It provides technical assistance to improve healthcare systems, disease surveillance, and health policy implementation. In emergencies, it leads international responses to pandemics, natural disasters, and humanitarian crises, tailoring its engagement to each country’s specific needs and capacities [2]. During pandemics, the WHO provides clear and reliable guidance to countries on the implementation of emergency response mechanisms. The organization issues binding instruments (such as the International Health Regulations) as well as recommendations, technical information, protocols, and best practices for establishing systems to track and monitor the spread of pathogens and to collect epidemiological data on infection rates [3].

The year 2020 revealed that many health systems were unprepared, underfunded, and understaffed to effectively respond to the emergence of the novel coronavirus that caused the COVID-19 pandemic [4]. This resulted in a decline in vaccination rates, deterioration in maternal and perinatal care, and interruptions to cancer prevention and screening programs [5,6]. The World Health Organization (WHO) collaborated with national governments to establish effective emergency coordination mechanisms in response to the COVID-19 pandemic. Educating the public about the importance of hand hygiene, mask-wearing, physical distancing, and other preventive behaviors was one of the WHO’s key strategies to protect individuals from contracting COVID-19 and to mitigate further transmission of the virus [7]. European countries introduced lockdowns and formulated sanitation guidelines in accordance with the recommendations of the WHO [8]. In addition, the WHO provides comprehensive guidance on the clinical management of COVID-19 patients, with a particular focus on the treatment of severe cases, ventilator use, and intensive care protocols [6]. To date, the WHO has published 31 clinical guidelines related to the treatment and prevention of COVID-19. The first guideline on the clinical management of the novel coronavirus titled *Clinical management of severe acute respiratory infection when novel coronavirus (nCoV) infection is suspected*, was released on 12 January 2020. The clinical care documents have been continuously published and revised to reflect emerging evidence. The Clinical Management of COVID-19 guideline has undergone seven updates, the most recent of which occurred on 15 July 2025. Similarly, the Therapeutics and COVID-19 guideline, which provides recommendations for the use of pharmacological treatments, has been updated fourteen times [9].

One of the major challenges in healthcare systems during the COVID-19 pandemic was the shortage of personal protective equipment (PPE) for medical personnel, including masks, gowns, gloves, and face shields. In response to these shortages, the World Health Organization (WHO) issued interim guidance titled “Rational use of personal protective equipment for COVID-19 and considerations during severe shortages.” The document was released in four versions: the first version on 27 February 2020, providing initial guidelines on the rational use of PPE in the context of COVID-19; the second version on 19 March 2020; the third on 6 April 2020; and the final version on 23 December 2020, updating the guidelines to incorporate new experiences gained during the pandemic [10]. The final version of the document consolidates WHO recommendations on the rational use of PPE in healthcare settings and outlines temporary strategies to be employed during acute supply shortages. The document also includes two annexes: Annex 1 provides updated PPE use recommendations for healthcare workers based on transmission scenario, setting, and activity within the context of COVID-19, while Annex 2 presents updated considerations for the decontamination or reprocessing of PPE. This guidance is intended for public health authorities, organizations, and designated personnel involved in decision-making regarding PPE distribution, management, and utilization by healthcare workers [10].

In this study, we focus on the WHO interim guidance introduced on 23 December 2020, entitled “Rational use of personal protective equipment for COVID-19 and considerations during severe shortages.” This document is partly based on scientific evidence, expert analyses, and clinical and operational experience. In its publications, the WHO draws on international experience, particularly from countries that have previously faced outbreaks of SARS and MERS. The organization relies on the expertise of specialists in infection prevention and control, infectious diseases, epidemiology, and protective equipment engineering, who evaluate benefits and risks and develop recommendations grounded in current evidence and resource availability. Furthermore, the WHO document addresses the issue of PPE shortages and outlines contingency strategies that require systemic analysis, such as logistics and resource allocation, to ensure that the recommendations are practical and applicable across different national contexts and levels of healthcare systems. To the best of our knowledge, no studies have yet evaluated the usefulness of the WHO guidelines issued during the COVID-19 pandemic from the perspective of healthcare workers. Consequently, we undertook this assessment.

To situate this study within the broader European context, it is important to clarify the relevance of examining the implementation of the WHO guidance in Poland. Poland represents a valuable case study in the European landscape because its healthcare system experienced significant strain during the pandemic while attempting to maintain continuity of care. International evidence indicates that healthcare workers globally bore a disproportionately high burden of COVID-19 infections and deaths among medical personnel [11]. Reports from Poland documented substantial disruption of medical facilities and service delivery across regions during the pandemic [12]. Investigating the awareness and implementation of the WHO PPE guidance in this setting provides insight into how international recommendations were translated into practice under real-world constraints.

This study was informed by implementation research, which emphasizes that the effectiveness of international public health recommendations depends on their awareness, local implementation, and perceived usefulness in everyday practice. The objectives were to assess healthcare workers’ awareness of the WHO guidance on the rational use of personal protective equipment (PPE), to examine its institutional implementation, and to explore perceptions of its impact on workplace safety and preparedness among surveyed healthcare workers in Poland.

## 2. Materials and Methods

### 2.1. Target Study Group

A questionnaire-based study was directed at healthcare personnel who worked within both the public and private healthcare systems during the COVID-19 pandemic and in the subsequent period. Due to the large number of active medical facilities registered in Poland (1214 hospitals as of February 2025), a target study group constituting 20% of all entities was randomly selected. This resulted in 243 hospitals being invited to participate in the study. For this purpose, data from the System of Basic Hospital Service Provision (PSZ) were used. The PSZ was introduced in Poland in 2017 to ensure stable and continuous access to publicly funded healthcare services. This system encompasses medical facilities of varying levels of reference, ranging from county hospitals to clinical centers, thereby creating a hierarchical structure of hospital care [13]. The names of individual healthcare facilities from the complete list of PSZ-registered hospitals in Poland were numbered. Subsequently, using a random number generator [14], 20% of the units were selected (a total of 243 records were generated). For each selected facility, a general email address was identified to which the questionnaire-based study was sent. Among the randomly selected medical units, hospitals from first- to fourth-level reference were included.

### 2.2. Questionnaire-Based Study

The survey was conducted among healthcare workers employed at medical facilities selected for the target study group. Participation was voluntary and anonymous, and participants were informed that completion of the questionnaire constituted informed consent to participate. No identifying personal data (such as name or surname) were collected, and participants could withdraw at any time without consequence. The questionnaire used in this study was specifically developed to assess awareness and perceived usefulness of the WHO document within Polish healthcare settings. The initial version of the questionnaire was reviewed for content validity by five independent experts in clinical epidemiology, public health, and hospital management. The questionnaire was not intended as a psychometric instrument but as a purpose-designed survey tool to descriptively assess awareness, institutional implementation, and perceived usefulness of the WHO PPE guidance. As such, formal reliability testing or construct validation (e.g., factor analysis, internal consistency) was not performed. Following their recommendations, minor modifications were made to enhance clarity, relevance of the items, and consistency with the structure of the WHO guidance.

The questionnaire consisted of 24 questions (the questionnaire form is provided in the Supplementary Materials in the original Polish language and in Table 1-in English). The questionnaire comprised three sections. The first section (1–5 questions) included general questions concerning age, gender, number of years of employment in a medical facility, the participant’s medical profession, and the type of medical facility where the participant most frequently works.

The second section (6–14) contained questions related to the pandemic period. The questions concerned awareness of the WHO document “Rational use of personal protective equipment for COVID-19 and considerations during severe shortages”, limitations in access to PPE, and training on the rational use of PPE within the institution. Participants were also asked: “What were the main difficulties in implementing the WHO recommendations in your workplace during the pandemic?” and “Did the application of WHO guidelines improve your sense of safety at work during the pandemic?”

The last section (questions 15–24) contained items related to the perceived effects of the WHO guidelines measured in 2025, as well as their potential applicability during future epidemics, environmental disasters, or military conflicts.

**Table 1:** Individual survey questions (English version).

General Questions	Pandemic Period	After the Acute Phases of Pandemic
What is your medical profession? Physician Nurse Paramedic Physiotherapist Medical Facility Administrative Staff Member Other (please specify)	Were you familiar with the WHO recommendations on the rational use of PPE from December 2020? Yes No I don’t remember	Do you currently continue to follow the WHO recommendations from 2020 regarding PPE use? Yes Partially No
How long have you been working in the medical profession? <5 years 5–10 years 11–20 years >20 years	How did you become acquainted with the content of this document? Professional training Through the employer Independent reading I have not become acquainted with it Other (please specify)	Do you believe that the 2020 WHO document contributed to improving the safety of healthcare personnel and patients in current medical practice? Strongly Strongly disagree agree 1 2 3 4 5
In what type of medical facility do you work most frequently? Hospital Primary Health Care Clinic (PHC) Specialist Medical Practice Private Practice Other (please specify)	Was the WHO document taken into account in the development of local or hospital procedures? Yes Partially No I don’t know	Did the WHO 2020 document influence current PPE procurement and management policies in your workplace? Yes No I don’t know
Age <25 years 25–40 years 41–55 years >55 years	Has your institution implemented the WHO recommendations from the above document? Yes Partially No I don’t know	Which changes to PPE procedures from the WHO 2020 document should, in your opinion, remain permanently implemented? Rational use of PPE Possibility of PPE reuse in case of supply shortages Implementation of local PPE use guidelines Implementation of nationwide PPE use guidelines Ensuring continuity of PPE supply chains Other (please specify)
Gender Female Male	How would you rate the availability of PPE (e.g., masks, gloves, gowns) in your workplace during the pandemic? Very poor Very good 1 2 3 4 5	Do you consider that the WHO 2020 document was a useful source of information in clinical practice? Yes Partially No
	Which PPE items were most often lacking in your workplace during the pandemic? (multiple answers possible) Surgical masks FFP2/FFP3 masks Face shields/goggles Gloves Protective gowns Other (please specify)	Did the WHO guidelines issued during the pandemic increase your awareness of the rational use of PPE? Yes, significantly Yes, slightly No
	Were training on the rational use of PPE organized in your workplace during the pandemic? Yes, regularly Yes, several times No I don’t know	Do you believe that the WHO 2020 document on rational PPE use remains relevant in the context of current medical practice? Yes Partially No
	Did applying the WHO guidelines improve your sense of safety in the workplace during the pandemic? Strongly Strongly disagree agree 1 2 3 4 5	How would you rate institutional support (e.g., from the Ministry of Health, WHO, Chief Sanitary Inspectorate) in implementing PPE recommendations? Very poor Very good 1 2 3 4 5
	What were the greatest difficulties in applying WHO recommendations in your workplace during the pandemic? Staff shortages Equipment shortages Lack of clear local guidelines Other (please specify)	Do you think WHO recommendations should be more closely adapted to local realities? Yes No I don’t know
		What are your general conclusions or suggestions regarding the use of PPE in future crisis situations (e.g., epidemics, natural disasters, anthropogenic disasters, armed conflicts, etc.)?

The questionnaires were distributed between 1 July 2025 and 31 August 2025. An email invitation to complete the survey was sent twice (at a one-month interval) to the general contact address of each selected medical facility. The forwarding or internal distribution of the survey link within institutions was not monitored. Of the 243 facilities invited, 44 (18.1%) actively declined participation, mostly citing organizational overload during the study period, and 199 (81.9%) did not provide an explicit response to the invitation. Responses were received from the 199 non-declining facilities through the open survey link between 1 July and 30 September 2025. Because the link allowed participation by any employee within the invited institutions, the number of eligible individual staff members is unknown, and a precise individual-level response rate cannot be calculated. At the facility level, the invitation-to-participation ratio corresponds to 542 responses from hospitals in the invited sample. Survey responses were collected until 30 September 2025.

Inclusion criteria were employment as a healthcare professional in an invited facility during the pandemic or after the acute phase of the pandemic (in 2025). Questionnaires with incomplete submissions could not be submitted through the survey platform and therefore no post-submission exclusions occurred. Flow summary: 1214 registered hospitals → 243 randomly selected → 243 invited → 44 declined → responses collected from the remaining invitation pool → 542 individual completed questionnaires.

### 2.3. Data Analysis

The survey results were converted into percentages based on the responses obtained. Additionally, a more in-depth analysis was performed for selected questions. The responses were sorted according to profession, direct contact with patients or lack thereof, and professional groups, as well as by sex and age. Percentages of answers were calculated within each respective group.

## 3. Results

### 3.1. Participants

**Figure 1:**
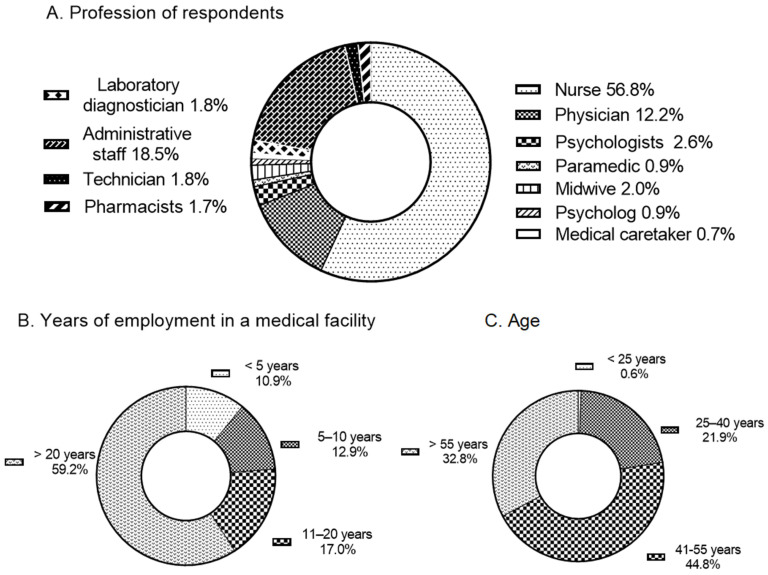
Characteristics of the study participants. (**A**) shows the occupational distribution of respondents, including personnel with and without direct patient contact. (**B**) presents the age distribution of the study group. (**C**) displays the number of years respondents had been employed in medical facilities, reflecting the overall seniority structure of the sample.

A total of 542 individuals participated in the study. The majority of responses were provided by personnel directly involved in patient care, including nurses (56.8%), physicians (12.2%), physiotherapists (2.6%), paramedics (0.9%), and midwives (0.9%), collectively accounting for approximately 75% of all responses. The remaining 25% were submitted by personnel not directly engaged in patient care. Within this group, the largest proportion of all respondents consisted of administrative staff of medical institutions (18.5%), laboratory diagnosticians (1.8%) and technicians (1.8%) (Figure 1A).

The majority of respondents were women (86.8%). The survey was most frequently completed by middle-aged medical personnel (41–55 years; 44.7%) and older personnel (over 55 years; 32.8%), typically possessing extensive professional experience. The largest subgroup comprised individuals who had been employed in healthcare institutions for more than 20 years (59.3%). Detailed characteristics of the study population are presented in Figure 1B,C.

### 3.2. Responses Related to the Period of the COVID-19 Pandemic

When asked whether they were familiar with the WHO recommendations on the rational use of PPE issued in December 2020, 76.5% of respondents answered affirmatively. A negative response was provided by 7.1% of participants, while the remainder could not recall this document. Among healthcare professionals with direct patient contact (e.g., physicians, nurses, physiotherapists), more than 78% reported familiarity with the WHO guidelines. A slightly lower proportion was observed among personnel without direct patient contact (65.9%), who more frequently stated that they could not recall the document (25.0% vs. 12.9%). Direct-care personnel showed higher familiarity with the December 2020 WHO recommendations on rational PPE use (80.3%) than non-direct staff (65.1%), although awareness varied widely across professions, with physiotherapists, nurses, and medical caretakers scoring highest and paramedics and psychologists showing the largest gaps. Non-direct staff demonstrated lower overall familiarity and greater uncertainty, with laboratory diagnosticians showing full awareness, while administrative staff, technicians, and especially pharmacists most often reported incomplete knowledge or not remembering the guidance.

The subsequent question concerned the source of familiarity with the WHO document. Nearly half of the respondents indicated that they had become acquainted with it through their employer (48.6%). The second most frequently selected response (22.6%) was individual reading of the document. Lack of familiarity with the document was declared by one in ten employees of medical institutions (10.2%). Direct-care staff most often became acquainted with the WHO document through their employer (47.2%), followed by independent reading (22.6%) and professional training (18.9%), with only 8.8% reporting no acquaintance; nurses showed a nearly identical pattern (47.0% employer, 23.4% independent reading). Physiotherapists relied heavily on the employer (71.4%) but also had comparatively high rates of not becoming acquainted with the document (14.3%). Among non-direct staff, the employer was likewise the primary source (50.8%), but they showed much lower participation in professional training (4.7%), higher rates of no acquaintance (14.1%), and more frequent reliance on “other” sources (8.6%).

Approximately 60% of respondents reported that the WHO document had been taken into account in the development of local or hospital procedures for the use of PPE. Around 10% acknowledged that the guidelines had been partially implemented. A further 28.2% of respondents selected the option “I do not remember”, while only 1.5% stated “no.” Direct-care staff showed clearly higher awareness and implementation of the WHO PPE guidance, with 64.8% reporting full consideration of the document and substantially fewer answering “I don’t know” compared with non-direct staff, among whom uncertainty reached nearly 50%. The highest incorporation of the WHO recommendations was observed among midwives, medical caretakers, physiotherapists, and nurses, whereas non-direct groups—especially laboratory diagnosticians, technicians, administrative staff, and pharmacists—most often reported limited awareness or uncertainty regarding the use of the document in local procedures. A similar distribution of responses was obtained for the question regarding whether the institution had implemented the WHO recommendations from the aforementioned document. 63.1% of respondents answered “yes,” 13.1% indicated “partially,” 23.1% selected “I do not know,” and only 0.7% responded “no.” Among direct-care staff, 64.8% reported full consideration of the WHO document (11.2% partially, 1.2% not), compared with only 42.7% among non-direct personnel, who also showed much higher uncertainty (49.2% vs. 22.7%). The highest incorporation was seen in midwives (81.8%), medical caretakers (80%), physiotherapists (71.4%), and nurses (67.9%), while laboratory diagnosticians (70%) and technicians (50%) led among non-direct staff, and administrative workers as well as most pharmacists most often reported not knowing whether the document had been used.

**Figure 2:**
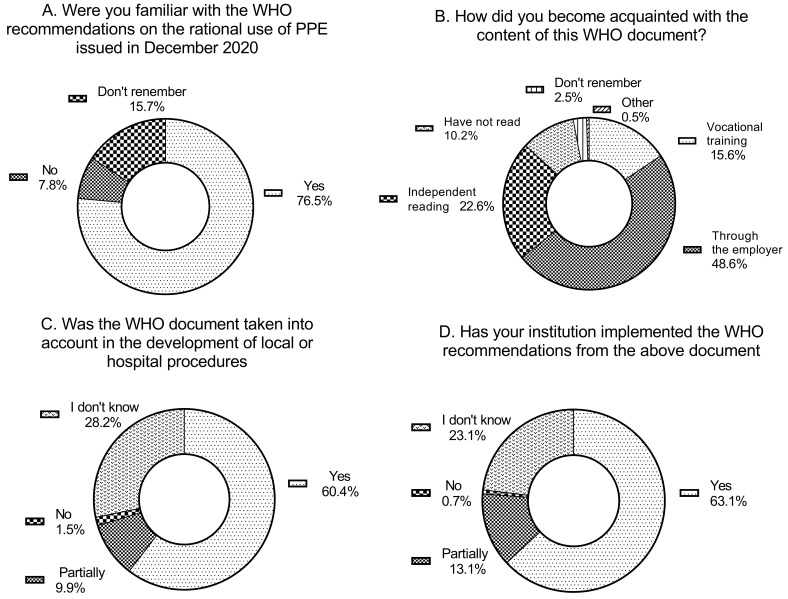
Responses describing awareness and institutional implementation of the WHO guidance on rational use of personal protective equipment during the COVID-19 pandemic. (**A**) shows the proportion of respondents who were familiar with the WHO document. (**B**) presents the reported sources of information about the document. (**C**) displays whether the WHO guidance was taken into account in the development of local or hospital procedures. (**D**) illustrates whether respondents believed that their institutions had implemented the WHO recommendations.

The described data are presented in Figure 2A–D and in Table 2A–D.

In response to the question about the availability of personal protective equipment during the pandemic, the majority of respondents rated it as very good and good, at 54% and 28.6%, respectively. Only just under 7% of respondents reported that availability was poor or very poor. Personnel directly involved in patient care, slightly worse than personnel not directly engaged in patient care, rated the availability of PPE during the pandemic (7% of the former rated it as “Very poor” or “Poor” as compared to 2.4% percent of the latter). In turn, the availability of PPE somewhat more frequently was perceived as “Good” or “Very good” by nurses as compared to physicians (85.7% vs. 73.9%).

When asked whether training sessions on the rational use of PPE were organized in their workplace during the COVID-19 pandemic, 40.1% of respondents reported that such training had been conducted several times, and 23.3% indicated that it had been held regularly. Only 19% of participants stated that no such training had been organized. Regarding the question about the training on the rational use of PPE, a noticeably higher percentage of personnel involved in patient care than non-medical staff confirmed that they were organized in their workplace (76.4% vs. 53.2%). In this respect, the difference between nurses and physicians was minimal (67.9% and 63.1%, respectively).

The following two questions addressed the availability of PPE during the COVID-19 pandemic. Respondents most frequently reported shortages of FFP2/FFP3 masks (61.6%). At a comparable level, shortages were noted for face shields or goggles (20.0%), protective gowns (18.3%), and surgical masks (16.3%). Interestingly, one in five respondents declared that they had not experienced any shortages of PPE during the pandemic.

The final questions concerning the pandemic period addressed respondents’ subjective assessment of the impact of the WHO recommendations on improving their sense of safety in the workplace, as well as their views on the main difficulties encountered in implementing the WHO guidelines at their institutions during the pandemic. In the first question, the vast majority of respondents provided a positive evaluation, a total of 68.0% perceived safety associated with the WHO recommendations rated as 4 or 5 on a five-point scale. A significant majority of both personnel actively engaged (68.7%) and not engaged in patient care (64.7%) acknowledged that applying the WHO guidelines improved their sense of safety in the workplace. Interestingly, this percentage was clearly higher among nurses (72.0%) compared to physicians (54.5%).

When asked about the greatest difficulties in implementing the WHO recommendations, respondents most frequently indicated staff shortages (52.9%), followed by equipment shortages (17.6%), and a lack of clear local guidelines (20.6%). As regards the greatest perceived difficulties in applying the WHO recommendations in the workplace, staff shortages were definitely the most commonly mentioned by both workers involved (52.3%) and those not involved in patient care (43.4%). This obstacle was more frequently identified by nurses (55.7%) than by physicians (40.0%). Other difficulties, such as, for example, equipment shortages or lack of clear local guidelines, were indicated by respondents much less often.

**Figure 3:**
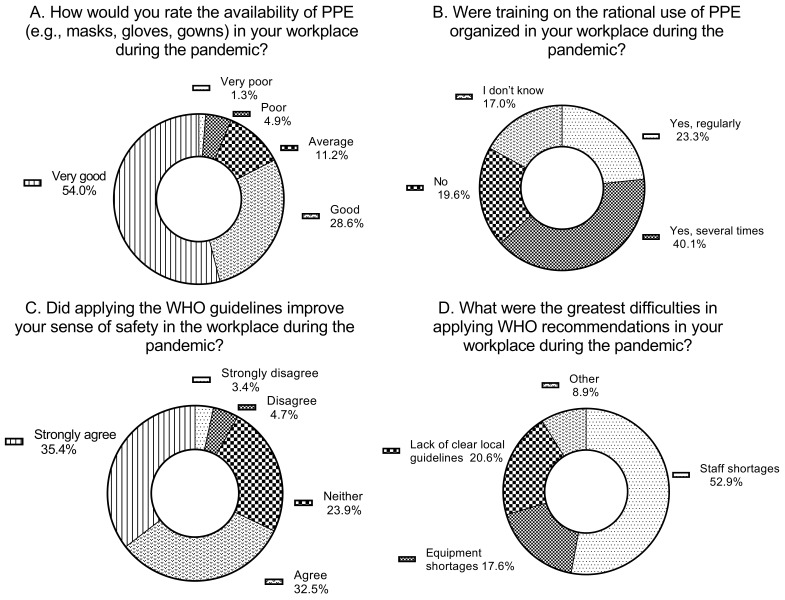
Responses describing training activities, availability of personal protective equipment, and perceived effects of WHO recommendations during the COVID-19 pandemic. (**A**) presents respondents’ ratings of the availability of personal protective equipment (PPE), such as masks, gloves, and gowns, in their workplace during the pandemic. (**B**) shows whether training on the rational use of PPE was organized in the workplace. (**C**) illustrates respondents’ ratings of how the WHO guidance influenced their sense of safety at work. (**D**) identifies the main difficulties encountered in applying the recommendations.

The described data are presented in Figure 3A–D and in Table 3A–D.

### 3.3. Responses Related to the Period After the COVID-19 Pandemic

The next set of questions concerned the period following the pandemic. More than half of the respondents (52.3%) reported continued adherence to the 2020 WHO recommendations on the use of PPE, while 41.2% indicated that they partially adhere to these guidelines. The remaining participants declared that they do not apply the WHO recommendations in their current professional practice. The adherence was higher among nurses (58.6% adhered and 37.8% partially adhered) than physicians (42.4% and 39.4%, respectively). Nearly one in five physicians stated that they no longer adhere to the WHO PPE recommendations. Interestingly, the highest adherence was observed in midwives, which may be related to the health concerns about infants.

A total of 70.8% of respondents stated that the WHO document continues to enhance the safety of healthcare personnel and patients in current medical practice (ratings of 4 or 5 on a five-point scale). Furthermore, 41.2% of participants indicated that the WHO guidelines influence current procurement and PPE management policies in their workplaces, whereas approximately one in five respondents believed that the document has no such impact. There was no visible difference in opinion between personnel directly involved in patient care and not engaged, but physicians (53.8%) tended to respond less positively than nurses (76.5%), following the general trend of more negative opinions on the document in the physician group.

When asked to identify the procedures that should currently be applied in healthcare facilities (multiple answers were allowed), respondents most frequently indicated rational use of PPE (53.7%) and the need to ensure continuity of PPE supply chains (44.2%). Interestingly, almost a third of respondents emphasized the importance of implementing nationwide (27.0%) and/or local (17.7%) recommendations. The majority of respondents confirmed that the WHO document was a useful source of information for clinical practice (57.3%). 59.1% of medical personnel viewed this document as a useful source, 35.5% as partially useful, and only 5.4% didn’t find it helpful. Most of the personnel stated that the document enhanced their awareness regarding the rational use of PPE (61.5%). The stated increase in awareness was higher in personnel involved in patient care (63.6%) than in non-medical staff (53.7%). The dynamics of awareness were lower in physicians than in nurses. 16.7% of physicians responded that the WHO guidelines didn’t increase their awareness of the rational use of protective equipment, while this statement was made by only 5% of nurses. Most participants affirmed the continued relevance of this document in the context of current healthcare practice (58.7%).

**Figure 4:**
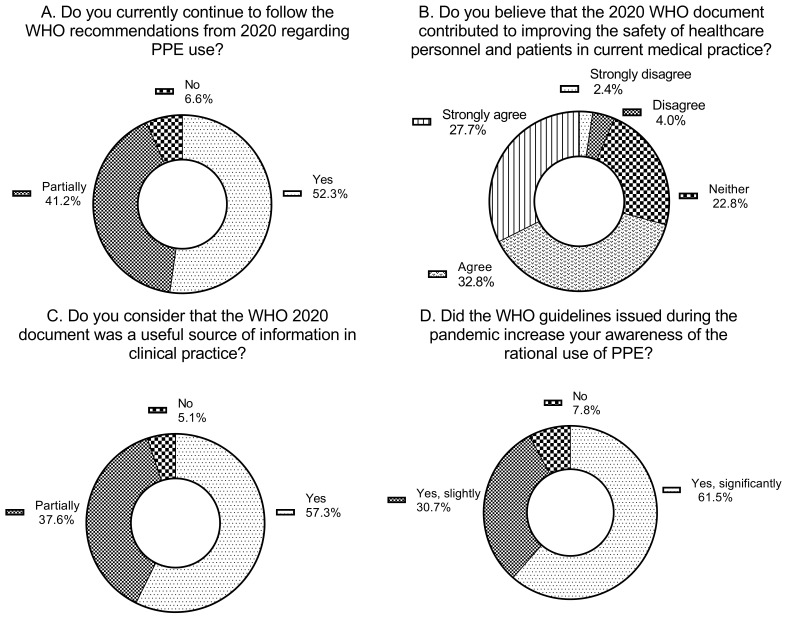
Responses related to the use and perceived usefulness of the WHO guidance in 2025 (after the acute phase of the pandemic). (**A**) shows whether respondents continue to follow the 2020 WHO recommendations in current practice. (**B**) presents the extent to which the guidance is believed to improve the safety of healthcare personnel and patients after the pandemic. (**C**) illustrates whether the recommendations have influenced procurement and management policies for personal protective equipment. (**D**) indicates which procedural elements from the WHO document respondents believe should remain permanently in place.

The described data are presented in Figure 4A–D and in Table 4A–D.

When asked about institutional support, for example, from the Polish Ministry of Health, the WHO, or the Chief Sanitary Inspectorate, in implementing PPE-related recommendations, only 34.5% of respondents rated this support as good, and 13.6% as very good. The detailed responses between the groups with direct patient contact and those without such contact were at a similar level. Interestingly, the support was rated slightly worse by healthcare workers who had direct contact with patients. Moreover, approximately 80.2% of respondents believed that the WHO recommendations should be better adapted to local realities. Personnel directly involved in patient care answered this question positively in 81.5% of cases. Comparable results were observed among personnel not directly engaged in patient care, with 76.4% positive responses. No significant or distinct differences were noted between the groups in the proportion of negative responses.

**Table 4:** Responses related to the use and perceived usefulness of the WHO guidance in 2025 (after the acute phase of the pandemic), incorporating the nature of patient contact and variations across professional groups. Panel A shows whether respondents continue to follow the 2020 WHO recommendations in current practice. Panel B presents the extent to which the guidance is believed to improve the safety of healthcare personnel and patients after the pandemic. Panel C illustrates whether the recommendations have influenced procurement and management policies for personal protective equipment. Panel D indicates which procedural elements from the WHO document respondents believe should remain permanently in place.

A. Do you currently continue to follow the WHO recommendations from 2020 regarding PPE use?	**Medical Roles**	**Yes**	**Partially**		**No**		**Number of Answers**
	**Personnel directly involved in patient care**	**56.2%**	**37.4%**		**6.4%**		**406**
	Nurses	58.6%	37.8%		3.6%		304
	Physicians	42.4%	39.4%		18.2%		66
	Physiotherapists	42.9%	50.0%		7.1%		14
	Paramedics	25.0%	50.0%		25.0%		4
	Midwives	90.9%	9.1%		-		11
	Psychologists	33.3%	33.3%		33.3%		3
	Medical caretake	80.0%	20.0%		-		5
	**Personnel not directly engaged in patient care**	**39.5%**	**53.2%**		**7.3%**		**124**
	Administrative staff of medical institutions	37.8%	54.1%		8.2%		98
	Laboratory diagnosticians	70.0%	30.0%		-		10
	Technicians	33.3%	66.7%		-		9
	Pharmacists	28.6%	57.1%		14.3%		7
B. Do you believe that the 2020 WHO document contributed to improving the safety of healthcare personnel and patients in current medical practice?	**Medical Roles**	**Strongly Disagree**	**Disagree**	**Neither**	**Agree**	**Strongly Agree**	**Number of Answers**
	**Personnel directly involved in patient care**	**2.5%**	**4.0%**	**21.7%**	**37.8%**	**34.1%**	**405**
	Nurses	2.3%	3.9%	17.3%	39.9%	36.6%	306
	Physicians	3.1%	4.6%	38.5%	30.8%	23.1%	65
	Physiotherapists	7.1%	-	42.9%	28.6%	21.4%	14
	Paramedics	-	-	-	66.7%	33.3%	3
	Midwives	-	-	36.4%	27.3%	36.4%	11
	Psychologists	-	-	50.0%	-	50.0%	2
	Medical caretake	-	20.0%	0.0%	40.0%	20.0%	5
	**Personnel not directly engaged in patient care**	**2.4%**	**4.0%**	**26.6%**	**41.1%**	**25.8%**	**124**
	Administrative staff of medical institutions	3.1%	1.0%	28.6%	43.9%	23.5%	98
	Laboratory diagnosticians	-	10.0%	20.0%	20.0%	50.0%	10
	Technicians	-	-	22.2%	44.4%	33.3%	9
	Pharmacists	-	42.9%	14.3%	28.6%	14.3%	7
C. Do you consider that the WHO 2020 document was a useful source of information in clinical practice?	**Medical Roles**	**Yes**	**Partially**		**No**		**Number of Answers**
	**Personnel directly involved in patient care**	**59.1%**	**35.5%**		**5.4%**		**406**
	Nurses	60.3%	35.4%		4.3%		305
	Physicians	56.1%	33.3%		10.6%		66
	Physiotherapists	57.1%	35.7%		7.1%		14
	Paramedics	66.7%	33.3%		-		3
	Midwives	54.5%	45.5%		-		11
	Psychologists	-	50.0%		50.0%		2
	Medical caretake	-	40.0%		60.0%		5
	**Personnel not directly engaged in patient care**	**50.4%**	**45.5%**		**4.1%**		**121**
	Administrative staff of medical institutions	49.0%	47.9%		3.1%		96
	Laboratory diagnosticians	80.0%	20.0%		-		10
	Technicians	44.4%	44.4%		11.1%		9
	Pharmacists	33.3%	50.0%		16.7%		6
D. Did the WHO guidelines issued during the pandemic increase your awareness of the rational use of PPE?	**Medical Roles**	**Yes, Significantly**	**Yes, Slightly**		**No**		**Number of Answers**
	**Personnel directly involved in patient care**	**63.6%**	**29.0%**		**7.4%**		**404**
	Nurses	65.2%	29.8%		5.0%		302
	Physicians	60.6%	22.7%		16.7%		66
	Physiotherapists	42.9%	50.0%		7.1%		14
	Paramedics	-	100.0%		-		3
	Midwives	72.7%	27.3%		-		11
	Psychologists	33.3%	-		66.7%		3
	Medical caretake	80.0%	-		20.0%		5
	**Personnel not directly engaged in patient care**	**53.7%**	**37.2%**		**9.1%**		**121**
	Administrative staff of medical institutions	56.3%	36.5%		7.3%		96
	Laboratory diagnosticians	50.0%	40.0%		10.0%		10
	Technicians	36.4%	36.4%		9.1%		11
	Pharmacists	33.3%	33.3%		33.3%		6

**Figure 5:**
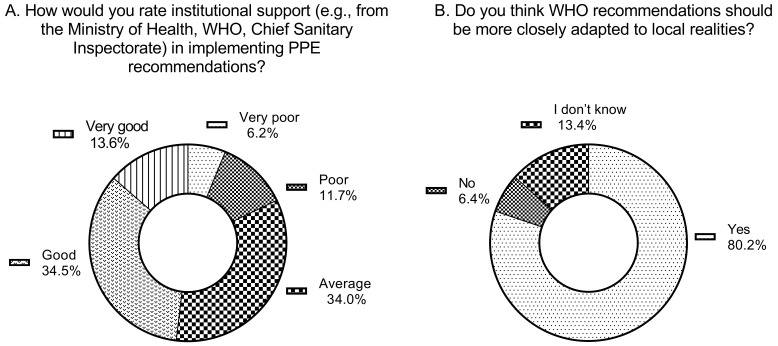
Responses describing perceptions of institutional support and general recommendations for future crisis preparedness. (**A**) shows respondents’ assessments of support received from national and international institutions in implementing personal protective equipment guidance. (**B**) summarizes the main suggestions provided by respondents regarding the use of personal protective equipment in future emergencies, including epidemics, natural disasters, and other crisis situations.

The final question was an open-ended item, asking participants for their general conclusions or suggestions regarding the use of PPE in future crisis situations, including epidemics, natural disasters, anthropogenic catastrophes, armed conflicts, and similar emergencies. Respondents emphasized the need for clear, practical, and locally adapted guidelines, supported by regular training for both medical and administrative personnel, as well as robust institutional coordination. Ensuring rapid, transparent information flow, centralized monitoring of PPE usage, and standardized procedures were repeatedly identified as essential for improving safety and preparedness. Finally, participants stressed that PPE must always be accessible in sufficient quantities, with ongoing education and implementation strategies grounded in real-world conditions rather than theoretical plans. The described data are presented in Figure 5A,B and Table 5A,B.

### 3.4. Analysis of Survey Responses by Gender and Age Groups

Additionally, we performed calculations regarding gender and age groups. We excluded the age group below 25 years, as it contained only three responses, which did not allow for reliable interpretation. The results for the remaining groups are presented in Table 6A–G. Overall, we did not observe major differences in the survey responses between gender and age groups; therefore, we included only the most interesting observations.

In the question assessing whether respondents were familiar with the WHO recommendations on the rational use of PPE from December 2020, we observed that awareness of this document increased with age from 68.6% in the 25–40 age group to 83.1% among respondents over 55 years old. This trend was also reflected in a decreasing proportion of individuals who were not familiar with the document. Interestingly, the methods by which the respondents learned about the document did not show any noticeable trends related to either gender or age. In the question concerning whether applying the WHO guidelines improved the respondents’ sense of safety in the workplace during the pandemic, women more frequently indicated that the guidelines enhanced their feeling of safety (70.3% compared with 49.3% of men). Conversely, men more often stated that the guidelines made no difference to their sense of safety (40.2% compared with 22% among women). Regarding the greatest difficulties in applying the WHO recommendations in the workplace during the pandemic, positive responses were reported more frequently by women than men (53.9% vs. 37.9%), while negative responses were more common among men (18.2% vs. 4.9%). Differences were also observed across age groups: younger respondents were less likely to report positive experiences compared to older participants (36.8% in the 25–40 age group vs. 64.6% in those over 50). The opposite trend was observed for negative responses in this question. In the question regarding institutional support—such as from the Ministry of Health, WHO, or the Chief Sanitary Inspectorate—women rated the support more positively than men, with 49.4% of women choosing “Good” or “Very good” compared to 36.7% of men.

## 4. Discussion

During the COVID pandemic, the WHO issued guidelines recommending a range of measures to be taken to mitigate the spread of the virus, including the promotion of hand hygiene, mask-wearing in public spaces, the maintenance of physical distancing, and the avoidance of mass gatherings. Similarly, WHO guidelines were issued for medical facilities. This was performed in order to limit the transmission of the virus [7,15]. The implementation of the WHO documents evoked diverse responses among society, which gradually evolved into more negative attitudes as the pandemic progressed [16]. Simultaneously, among healthcare workers, these documents were recognized as a guide for the effective execution of their professional responsibilities and for safeguarding themselves and their patients against viral transmission within hospital environments. The survey, completed by 542 healthcare workers, included sections on demographics and the perceived usefulness of the WHO document “Rational use of personal protective equipment for COVID-19 and considerations during severe shortages” during and after the pandemic. Participants, both with and without direct patient contact, were mostly over 40 years old and had over 20 years of professional experience. This roughly corresponds to the distribution of medical personnel in Poland, as reported in previous studies, while acknowledging the descriptive nature of the present sample. In a study on employment trends among healthcare workers with direct patient contact conducted by Małyszko et al. (2024), it was shown that in 2022, among physicians, 64.5% of employees were over 44 years of age, while in the group of nurses, this proportion reached as high as 83.5% of respondents [17]. Similar employment trends are also observed in other European Union countries. In the Eurostat report “Medical Workforce in the EU: An Ageing Profession” (2025), it was shown that the medical workforce is rapidly ageing, with 12 EU member states reporting that the proportion of physicians aged 55 years and over exceeded 40.0% in 2022 [18]. Despite the responders being mostly middle-aged and older and having extensive professional experience, self-reported familiarity (76.5%) with the WHO guidelines was high. Gordon et al. (2024) show that older adults may face barriers in accessing digital information, while Glass et al. (2015) highlight that age and prior experience influence willingness to participate in research [19,20]. Our findings suggest that professional engagement and workplace dissemination compensated for potential age-related limitations, though self-selection likely contributed to the high reported awareness, with younger or less experienced staff potentially underrepresented.

This relatively high awareness of the WHO-issued legal and procedural guidelines appears to be directly related to the emergence of a new global threat, which motivated individuals to seek protection by consulting the most reliable and authoritative sources. Similar findings have been reported by other researchers who assessed the knowledge of the clinical presentation, management, and diagnostic procedures related to COVID-19 among medical staff. In a survey conducted by Ahmed et al. among 810 healthcare professionals, more than half (52%) were aware of the COVID-19 guidelines, and 72% reported adherence to adequate preventive measures, although the majority (73%) had not participated in any training or workshop on the subject [21]. Also, in Saudi Arabia, research conducted on 957 healthcare workers, mostly female nurses aged 31–40, found that knowledge of COVID-19 was high (mean 9.89/12), wherein 58% attended simulations, and nearly all had N95 fit testing [22].

In our study, almost 50% of respondents stated that their knowledge of the referenced WHO document had been provided by their employer, while approximately 20% reported obtaining this information independently. These findings reflect a high level of knowledge dissemination by employers, a trend also observed in other European countries. In a study describing the availability of personal protective equipment (PPE) in 19 European countries [23], it was shown that in 17 of them, training materials or instructions on PPE use were publicly available. In eight countries, mandatory training for healthcare workers on the use of PPE was required in certain settings. The analysis also revealed that despite the availability of educational materials, there were still substantial differences between countries in terms of overall preparedness and access to protective equipment. Interestingly, the authors highlighted that the most prepared countries for the availability and use of PPE, measured in the PPE-preparedness index, were Ireland and Poland. These observations are consistent with the findings reported by respondents of our survey, where most respondents assessed PPE availability during the pandemic as good (28.6%) or very good (54.0%). Similar data were reported in another survey conducted among nurses in Poland. In that study, 69.0% of nurses stated that PPE was supplied “sometimes,” 29.3% reported that it was “always” available, and 1.7% indicated that PPE was not available in their workplace [24]. In the referenced study, the nurses most frequently reported shortages of masks (78%), protective coveralls (68.3%), and goggles or shoe covers (52.4%). In our survey, 78.4% of respondents also reported a lack of masks (both FFP2/3 and surgical), while 20% indicated shortages of goggles or face shields, and 18.3% reported insufficient availability of protective gowns.

The results obtained in the study revealed that, according to the respondents, many healthcare facilities (60%) incorporated the WHO recommendations into local procedures. However, these findings reflect subjective assessments rather than confirmed institutional practices. Additionally, healthcare facility managers organized training sessions on the rational use of PPE during the pandemic, with 40.1% of respondents confirming participation in regular educational activities. Regarding the use of PPE as reported by surveyed healthcare workers in Poland in the context of the WHO guidelines, 32.5% of respondents agreed, and 35.5% strongly agreed that the issued WHO recommendations improved their sense of workplace safety, while the main difficulties in adhering to the guidelines were staff shortages (52.9%) and lack of detailed local protocols (20.6%).

Collaboration and coordination are fundamental in today’s interconnected world. Teams that coordinate effectively are able to integrate diverse perspectives, skills, and resources to achieve superior outcomes. Effective collaboration enhances problem-solving capacity, fosters innovation, and strengthens the ability to adapt rapidly to change. Coordinated action is particularly vital in times of crisis, when swift and unified responses can save lives and protect livelihoods [25]. The COVID-19 pandemic profoundly affected multiple dimensions of human life and work, prompting unprecedented global collaboration to address the crisis. International efforts spanned numerous domains, including diagnostics, vaccine and treatment development, viral genome sequencing, and clinical research [26]. In our survey, respondents also evaluated the implementation process of the WHO interim guidance titled “Rational use of personal protective equipment for COVID-19 and considerations during severe shortages.” The assessment captured respondents’ perceptions of implementation at both the local level (decisions made by hospital directors or facility owners) and the national level (decisions made by the Ministry of Health, the Chief Sanitary Inspectorate, and related authorities). A total of 60.4% of respondents reported that the aforementioned WHO document was taken into account when developing local or hospital procedures. A comparable proportion (63.1%) indicated that their medical institutions had implemented the recommendations outlined in the WHO guidance. Interestingly, more than half of the respondents (52.3%) reported continued adherence to the 2020 WHO recommendations regarding the rational use of personal protective equipment (PPE). This is also reflected in the overall positive evaluation of the WHO document—61.5% of respondents agreed that it contributed to increased awareness regarding the rational use of PPE, and 58.7% considered the document to remain relevant in their current professional practice.

Although the present study demonstrates a relatively high level of familiarity with the WHO document “Rational use of personal protective equipment for COVID-19” and considerations during severe shortages among respondents, these findings reflect self-reported perceptions rather than objectively verified institutional practices. The coexistence of high individual awareness and only moderate ratings of support from national institutions (e.g., the Ministry of Health, WHO, and the Chief Sanitary Inspectorate) suggests a meaningful discrepancy between personal engagement with the WHO guidance and the systemic conditions required for its practical implementation. While 76.5% of respondents declared good familiarity with the recommendations and more than half (63.1%) reported that their facilities incorporated elements of the WHO guidance, only 13.6% assessed institutional support as very good, 34.5% as “good”, and 34 as “average”. This divergence aligns with observations from international studies, which indicate that global recommendations often face challenges when translated into local practice due to resource constraints, staff shortages, rapidly changing regulations, and limited operational capacity. Respondents in the present study similarly identified staff shortages, inconsistent PPE supply, and insufficiently detailed local protocols as barriers that hindered effective application of the WHO guidance. These factors may explain why healthcare workers simultaneously viewed the recommendations as relevant and valuable yet perceived the institutional support framework as only moderate. Although available public sources and respondents’ reports suggest that Polish governmental institutions and healthcare facilities did implement the WHO guidelines, or guidelines inspired by the WHO and other international organizations, particularly in the areas of prevention, diagnostics, and vaccination particularly in areas such as prevention, diagnostics, and vaccination—a complete, uniform implementation of all the WHO recommendations across healthcare settings in Poland was not comprehensively documented in publicly accessible sources. In addition, during the course of the pandemic, documentation and standards were frequently updated. Consequently, the implementation of the WHO guidance was a dynamic and iterative process rather than a one-time event. Hospitals and healthcare providers in Poland developed their own protocols, incorporating both WHO recommendations and national governmental regulations [27]. Despite these observations, it may be inferred that, with respect to this specific aspect of the WHO guidance, the Polish national health authorities acted in an appropriate and timely manner. On 31 March 2020, the National Institute of Public Health-National Institute of Hygiene (NIZP-PZH) also issued guidelines for diagnostic laboratories concerning COVID-19, explicitly stating that these recommendations followed the standards of the European Centre for Disease Prevention and Control (ECDC), which are indirectly aligned with the WHO guidance [28]. Also, the Medical Research Agency published information on the WHO Guidelines on Clinical Management of COVID-19, noting that the full text of the WHO document (Clinical management of COVID-19) was available and used as a national reference for clinical management practices in Poland [29].

The final question addressed the respondents’ evaluation of the WHO recommendations in the context of the realities of local medical facilities, as well as their suggestions regarding the use of PPE in future crisis situations (including epidemics, natural disasters, anthropogenic incidents, and armed conflicts). A large majority of respondents (80.2%) believed that the WHO guidelines should be better adapted to local conditions, while only 6.4% stated that no such adaptation was necessary.

The strength of this response, expressed by 80.2% of healthcare workers, demonstrates that the translation of global recommendations into practice remains a significant challenge. Our data indicate that the perceived misalignment is grounded in concrete systemic constraints. Respondents repeatedly pointed to staff shortages, inconsistent PPE supply, the absence of clear, facility-level operational protocols, and limited financial and infrastructural capacity as factors that limited the feasibility of applying the WHO guidance in its original form. Recent literature consistently demonstrates that global WHO recommendations often face substantial barriers when translated into local healthcare practice. Across diverse analyses, researchers have shown that the feasibility of implementing the WHO guidance depends not only on scientific evidence but on the complex, resource-dependent, and administratively constrained environments in which healthcare workers operate. Studies by Greenhalgh and Papoutsi, and Assefa et al., highlight recurrent gaps between global expectations and local realities, including shortages of personnel and supplies, limited governance capacity, and the need for improvised or modified procedures [30,31]. Similarly, Evans-Gilbert et al. (2024) [32] emphasize the necessity of considering local drug availability, healthcare system structure, and public health priorities when implementing international guidelines, while Pozzo and Virgili (2020) [33] highlight the need for locally tailored policies and culturally adapted communication, rather than relying solely on centralized guidance. These findings are consistent with our results, in which the vast majority of surveyed healthcare workers reported that the WHO recommendations required substantial adaptation to local conditions. Together, the evidence underscores that global guidelines must be complemented by context-sensitive strategies to ensure effective implementation in real-world health systems. These statements suggest that even guidelines that are technically sound and evidence-based may not achieve their intended effect when they fail to account for the structural and organizational variability across healthcare settings.

International research shows that barriers to implementing clinical or operational guidelines during the COVID-19 pandemic arose not only from organizational strain but also from significant uncertainty regarding legal implications for clinical decision making. In many countries, frontline healthcare workers expressed concern about possible legal consequences of decisions made under emergency conditions, particularly when operating under crisis standards of care, resource shortages, or altered protocols [34,35]. In the absence of clear legal protection or explicit liability shields, health professionals often reported anxiety about future malpractice claims or negligence lawsuits, which may influence their willingness to follow international or national guidance. This pattern suggests that, in addition to resource and organizational constraints, fear of legal accountability under uncertain medico-legal environments might have undermined adherence to recommended guidelines.

When viewed in the context of international analyses, the barriers reported by respondents in Poland show features consistent with challenges described in other healthcare systems. Staff shortages and variation in local operational procedures were reported as major obstacles to the application of the WHO guidance, and these factors have also been identified internationally as conditions that limit adherence to global recommendations. The wider environment of heightened legal scrutiny during the early stages of the pandemic likely contributed to a cautious approach to guideline implementation and may have intensified concerns about responsibility for decisions made under conditions of uncertainty. Although Poland did not introduce pandemic-specific legal protections for healthcare workers, international evidence suggests that apprehension regarding potential legal consequences was widespread across countries. This broader context indicates that the difficulties described by respondents were shaped not only by organizational and resource-related constraints but also by the legal and professional pressures experienced by healthcare personnel during the pandemic.

The operational difficulties reported by respondents also need to be considered within the wider disruption of routine healthcare pathways observed internationally during the COVID-19 pandemic. Studies from multiple health systems documented marked declines in preventive services and diagnostic activity, with delayed cancer diagnoses, reduced chronic disease monitoring, and interruptions to essential care for vulnerable patients [36,37]. These disruptions were associated with worsening clinical outcomes and raised concerns about the indirect burden of the pandemic on morbidity and mortality. Evidence from several countries also indicated an increase in medico-legal claims related to delayed or missed diagnoses and to altered standards of care arising from strained system capacity [38]. Situating the Polish findings within this context suggests that the difficulties in implementing the WHO recommendations were part of broader pressures on health system resilience, where shortages of personnel, inconsistent local protocols, and limited resources interacted with system-wide disruptions in care delivery.

The respondents’ suggestions regarding the use of PPE in future crisis situations can be summarized in ten main points. First, maintaining sufficient and stable PPE reserves at both the central and local levels improves the safety perception of healthcare personnel and patients during emergencies. Second, the use of PPE should be rational and appropriate, tailored to the type of hazard, level of exposure, and the needs of the medical facility to avoid shortages and waste. Third, regular training for medical staff, management, and administrative personnel is crucial, covering proper use, removal, and disposal of PPE. Fourth, clear, uniform, and practical procedures should be developed and implemented consistently across the healthcare system, taking into account the specific characteristics of each facility. Fifth, crisis teams should be designated at both national and local levels to manage the procurement, distribution, and oversight of PPE. Sixth, access to certified products should be guaranteed, along with the development of domestic or European PPE production to ensure quality and supply continuity. Seventh, fast information flow and educational campaigns targeted at both healthcare personnel and the general public should be conducted, including instructional videos, webinars, and visual materials. Eighth, guidelines and procedures should be adapted to the local realities of facilities, including financial, staffing, and spatial capacities, to ensure they are feasible in practice. Ninth, systems for monitoring PPE demand, consumption, and availability should be implemented to prevent overuse or critical shortages. Finally, psychological and ergonomic aspects, such as comfort, stress, communication, and proper sizing of PPE, should be considered to ensure effective protection and adherence to procedures. These conclusions synthesize the practical experiences of healthcare staff and indicate directions for improving the PPE system in future crisis situations.

Perceptions of safety reported by respondents should be interpreted in light of the substantial epidemiological burden experienced by healthcare workers during the first phase of the COVID-19 pandemic. International data indicate that frontline medical personnel faced a significantly higher risk of SARS-CoV-2 infection compared with the general population. In a large multicountry analysis, frontline health care workers were found to have a threefold higher probability of contracting COVID-19 and in some regions, health care workers accounted for up to twenty percent of confirmed cases [39]. Evidence from Italy further documented high infection rates and excess mortality among health care workers, particularly among general practitioners and community-based clinicians who were exposed before adequate protective measures were widely available [40]. These epidemiological realities suggest that the sense of increased safety reported by respondents was shaped not only by subjective impressions but also by the concrete recognition of occupational risk during the early phases of the pandemic.

It is also worth mentioning that research consistently highlights that healthcare workers’ perceptions and behaviours during the pandemic were shaped not only by the content of official guidance but also by the broader communication environment and levels of institutional trust. Studies show that communication styles and coherence of messaging directly influence trust in public health institutions and compliance with protective measures. For example, Yang and Huang (2021) demonstrated that inconsistent or highly interactive communication could undermine trust in scientific authorities, while clear, authoritative communication strengthened confidence in administrators [41]. Within clinical teams, effective communication was found to be essential for sustaining collaboration under crisis conditions [42]. Furthermore, longitudinal evidence indicates that satisfaction with governmental communication predicts changes in institutional trust over time [43], and experiences from Israel show that conflicting expert narratives can erode trust in pandemic governance [44]. These findings suggest that healthcare workers’ assessments of the WHO recommendations were likely affected by communication consistency and trust dynamics within the national pandemic response.

## 5. Limitations

The present study has several limitations. The voluntary nature of participation and the absence of monitoring for internal distribution may have led to self-selection bias. Healthcare workers with greater awareness of the WHO guidelines or stronger opinions about PPE implementation may have been more inclined to participate. Additionally, without facility-level response tracking, the geographic and institutional representativeness of the sample cannot be fully determined. The study relies on a cross-sectional design and self-reported perceptions. Without objective safety indicators, we cannot determine whether the improved safety perception of the participants reflects actual improvements in safety. Furthermore, as the questionnaire was purpose-designed and not validated as a psychometric instrument, formal reliability or construct validation was not performed, which constitutes an additional methodological limitation. Because the survey was distributed internally within invited institutions and the number of eligible employees was unknown, individual response rates could not be calculated. Therefore, the findings should be interpreted as descriptive and reflective of respondents’ perceptions rather than as representative of the Polish healthcare system as a whole. Future studies should aim for broader recruitment and higher participation rates to obtain a more balanced view of healthcare professionals’ perceptions.

## 6. Conclusions

The findings of this study suggest that the WHO guidance “Rational use of personal protective equipment for COVID-19 and considerations during severe shortages” was reported as implemented by healthcare workers in Polish facilities participating in the survey, particularly at the institutional level. More than half of healthcare workers who participated in the survey continue to adhere to these recommendations, highlighting their perceived relevance and usefulness in routine clinical practice beyond the pandemic period. Nevertheless, limited institutional support from national authorities and the need for better adaptation of global recommendations to local contexts were identified as key challenges in supporting effective implementation. The experience reported by surveyed healthcare workers in Poland indicates that the WHO documents can serve as a useful framework for crisis response; however, according to respondents’ assessments, their adoption depends on national coordination, clear communication, and regular professional training. Future preparedness strategies should therefore focus on considering the integration of international and national guidelines, promoting flexibility, rapid information flow, and continuity of PPE supply chains to enhance resilience in future health emergencies.

## Figures and Tables

**Table 2:** Responses outline the level of awareness and the extent of institutional implementation of the WHO guidance on the rational use of personal protective equipment (PPE) during the COVID-19 pandemic, incorporating the nature of patient contact and variations across professional groups. Panel A shows the proportion of respondents who were familiar with the WHO document. Panel B presents the reported sources of information about the document. Panel C displays whether the WHO guidance was taken into account in the development of local or hospital procedures. Panel D illustrates whether respondents believed that their institutions had implemented the WHO recommendations.

A. Were you familiar with the WHO recommendations on the rational use of PPE from December 2020?	**Medical Roles**	**Yes**	**No**	**Don’t Remember**			**Number of Answers**
	**Personnel directly involved in patient care**	**80.3%**	**7.1%**	**12.9%**			**411**
	Nurses	84.3%	5.2%	10.5%			306
	Physicians	63.6%	15.2%	21.2%			66
	Physiotherapists	85.7%	14.3%	-			14
	Paramedics	20.0%	40.0%	40.0%			5
	Midwives	72.7%	-	18.2%			11
	Psychologists	33.3%	-	66.7%			3
	Medical caretake	80.0%	-	20.0%			5
	**Personnel not directly engaged in patient care**	**65.1%**	**10.1%**	**24.0%**			**129**
	Administrative staff of medical institutions	63.1%	9.7%	26.2%			103
	Laboratory diagnosticians	100.0%	-	-			10
	Technicians	60.0%	10.0%	30.0%			10
	Pharmacists	57.1%	28.6%	14.3%			7
B. How did you become acquainted with the content of this document?	**Medical Roles**	**Professional Training**	**Through the Employer**	**Independent Reading**	**I have Not Become Acquainted with it**	**Other**	**Number of Answers**
	**Personnel directly involved in patient care**	**18.9%**	**47.2%**	**22.6%**	**8.8%**	**2.5%**	**407**
	Nurses	20.7%	47.0%	23.4%	6.9%	2.0%	304
	Physicians	16.7%	36.4%	22.7%	18.2%	6.1%	66
	Physiotherapists	-	71.4%	14.3%	14.3%	-	14
	Paramedics	-	60.0%	-	20.0%	20.0%	5
	Midwives	18.2%	63.6%	18.2%	-	-	11
	Psychologists	-	33.3%	-	33.3%	33.3%	3
	Medical caretake	20.0%	60.0%	20.0%	-	-	5
	**Personnel not directly engaged in patient care**	**4.7%**	**50.8%**	**21.9%**	**14.1%**	**8.6%**	**128**
	Administrative staff of medical institutions	3.9%	53.9%	16.7%	15.7%	9.8%	102
	Laboratory diagnosticians	50.0%	50.0%	-	-	-	10
	Technicians	10.0%	30.0%	40.0%	10.0%	10.0%	10
	Pharmacists	14.3%	28.6%	42.9%	42.9%	-	7
C. Was the WHO document taken into account in the development of local or hospital procedures?	**Medical Roles**	**Yes**	**Partially**	**No**	**I Don’t Know**		**Number of Answers**
	**Personnel directly involved in patient care**	**64.8%**	**11.2%**	**1.2%**	**22.7%**		**409**
	Nurses	67.9%	10.2%	0.7%	21.3%		305
	Physicians	48.5%	16.7%	1.5%	33.3%		66
	Physiotherapists	71.4%	14.3%	7.1%	7.1%		14
	Paramedics	40.0%	-	-	60.0%		5
	Midwives	81.8%	18.2%	-	-		11
	Psychologists	33.3%	-	-	66.7%		3
	Medical caretake	80.0%	-	20.0%	-		5
	**Personnel not directly engaged in patient care**	**42.7%**	**5.6%**	**2.4%**	**49.2%**		**124**
	Administrative staff of medical institutions	43.4%	6.1%	3.0%	47.5%		99
	Laboratory diagnosticians	70.0%	10.0%	-	20.0%		10
	Technicians	50.0%	-	-	50.0%		10
	Pharmacists	42.9%	-	-	57.1%		7
D. Has your institution implemented the WHO recommendations from the above document?	**Medical Roles**	**Yes**	**Partially**	**No**	**I Don’t Know**		**Number of Answers**
	**Personnel directly involved in patient care**	**67.7%**	**13.0%**	**0.5%**	**18.8%**		**409**
	Nurses	71.8%	12.1%	0.3%	15.7%		305
	Physicians	45.5%	21.2%	-	33.3%		66
	Physiotherapists	78.6%	7.1%	-	14.3%		14
	Paramedics	40.0%	0.0%	-	60.0%		5
	Midwives	90.9%	9.1%	-	-		11
	Psychologists	33.3%	-	-	66.7%		3
	Medical caretake	80.0%	-	20.0%	-		5
	**Personnel not directly engaged in patient care**	**47.2%**	**13.6%**	**1.6%**	**37.6%**		**125**
	Administrative staff of medical institutions	46.5%	13.1%	2.0%	38.4%		99
	Laboratory diagnosticians	70.0%	10.0%	-	20.0%		10
	Technicians	50.0%	10.0%	-	40.0%		10
	Pharmacists	28.6%	28.6%	-	42.9%		7

**Table 3:** Responses describing training activities, availability of personal protective equipment, and perceived effects of the WHO recommendations during the COVID-19 pandemic, incorporating the nature of patient contact and variations across professional groups. Panel A presents respondents’ ratings of the availability of personal protective equipment (PPE), such as masks, gloves, and gowns, in their workplace during the pandemic. Panel B (shows whether training on the rational use of PPE was organized in the workplace. Panel C illustrates respondents’ ratings of how the WHO guidance influenced their sense of safety at work. Panel D identifies the main difficulties encountered in applying the recommendation.

A. How would you rate the availability of PPE (e.g., masks, gloves, gowns) in your workplace during the pandemic?	**Medical Roles**	**Very Poor**	**Poor**	**Average**	**Good**	**Very Good**	**Number of Answers**
	**Personnel directly involved in patient care**	**1.5%**	**5.5%**	**11.4%**	**29.8%**	**53.3%**	**403**
	Nurses	1.3%	4.7%	9.6%	27.6%	58.1%	301
	Physicians	1.5%	9.2%	16.9%	38.5%	35.4%	65
	Physiotherapists	7.1%	14.3%	-	42.9%	42.9%	14
	Paramedics	-	20.0%	-	40.0%	40.0%	5
	Midwives	-	-	45.5%	9.1%	45.5%	11
	Psychologists	-	-	-	66.7%	33.3%	3
	Medical caretake	-	-	20.0%	20.0%	60.0%	5
	**Personnel not directly engaged in patient care**	**0.8%**	**1.6%**	**11.5%**	**27.0%**	**59.8%**	**122**
	Administrative staff of medical institutions	1.0%	1.0%	9.2%	28.6%	61.2%	98
	Laboratory diagnosticians	-	-	10.0%	20.0%	70.0%	10
	Technicians	-	-	12.5%	37.5%	50.0%	8
	Pharmacists	-	42.9%	28.6%	0.0%	28.6%	7
B. Were training on the rational use of PPE organized in your workplace during the pandemic?	**Medical Roles**	**Yes, Regularly**	**Yes, Several Times**	**No**	**I Don’t Know**		**Number of Answers**
	**Personnel directly involved in patient care**	**29.6%**	**46.8%**	**23.7%**	**14.9%**		**355**
	Nurses	25.6%	42.3%	21.0%	11.1%		305
	Physicians	26.2%	36.9%	24.6%	12.3%		65
	Physiotherapists	28.6%	21.4%	7.1%	42.9%		14
	Paramedics	20.0%	40.0%	40.0%	-		5
	Midwives	9.1%	54.5%	-	36.4%		11
	Psychologists	-	66.7%	-	33.3%		3
	Medical caretake	60.0%	20.0%	20.0%	0.0%		5
	**Personnel not directly engaged in patient care**	**15.9%**	**37.3%**	**16.7%**	**30.2%**		**126**
	Administrative staff of medical institutions	19.2%	37.4%	14.1%	29.3%		99
	Laboratory diagnosticians	10.0%	50.0%	20.0%	20.0%		10
	Technicians	-	50.0%	10.0%	40.0%		10
	Pharmacists	-	57.1%	0.0%	42.9%		7
C. Did applying the WHO guidelines improve your sense of safety in the workplace during the pandemic?	**Medical Roles**	**Strongly Disagree**	**Disagree**	**Neither**	**Agree**	**Strongly Agree**	**Number of Answers**
	**Personnel directly involved in patient care**	**3.7%**	**4.7%**	**23.0%**	**33.1%**	**35.6%**	**405**
	Nurses	2.6%	5.0%	20.5%	34.7%	37.3%	303
	Physicians	6.1%	6.1%	33.3%	24.2%	30.3%	66
	Physiotherapists	7.1%	-	14.3%	42.9%	35.7%	14
	Paramedics	-	-	-	33.3%	66.7%	3
	Midwives	-	-	45.5%	27.3%	27.3%	11
	Psychologists	33.3%	-	33.3%	33.3%	-	3
	Medical caretake	20.0%	-	20.0%	40.0%	20.0%	5
	**Personnel not directly engaged in patient care**	**2.5%**	**4.9%**	**27.9%**	**30.3%**	**34.4%**	**122**
	Administrative staff of medical institutions	1.0%	4.2%	30.2%	28.1%	36.5%	96
	Laboratory diagnosticians	-	10.0%	20.0%	30.0%	40.0%	10
	Technicians	10.0%	0.0%	20.0%	40.0%	30.0%	10
	Pharmacists	14.3%	14.3%	14.3%	57.1%	-	7
D. What were the greatest difficulties in applying WHO recommendations in your workplace during the pandemic?	**Medical Roles**	**Staff Shortages**	**Equipment Shortages**	**Lack of Clear Local Guidelines**		**Other**	**Number of Answers**
	**Personnel directly involved in patient care**	**52.3%**	**16.5%**	**18.5%**		**12.6%**	**405**
	Nurses	55.7%	15.3%	17.7%		11.3%	300
	Physicians	40.0%	20.0%	23.1%		16.9%	65
	Physiotherapists	42.9%	28.6%	21.4%		7.1%	14
	Paramedics	66.7%	16.7%	-		16.7%	6
	Midwives	45.5%	27.3%	18.2%		9.1%	11
	Psychologists	33.3%	-	33.3%		33.3%	3
	Medical caretake	80.0%	-	20.0%		-	5
	**Personnel not directly engaged in patient care**	**43.4%**	**17.1%**	**23.3%**		**16.3%**	**129**
	Administrative staff of medical institutions	43.7%	16.5%	25.2%		14.6%	103
	Laboratory diagnosticians	50.0%	20.0%	10.0%		20.0%	10
	Technicians	40.0%	30.0%	-		30.0%	10
	Pharmacists	28.6%	14.3%	42.9%		14.3%	7

**Table 5:** Responses describing perceptions of institutional support and general recommendations for future crisis preparedness, incorporating the nature of patient contact and variations across professional groups. Panel A shows respondents’ assessments of support received from national and international institutions in implementing personal protective equipment guidance. Panel B summarizes the main suggestions provided by respondents regarding the use of personal protective equipment in future emergencies, including epidemics, natural disasters, and other crisis situations.

A. How would you rate institutional support (e.g., from the Ministry of Health, WHO, Chief Sanitary Inspectorate) in implementing PPE recommendations?	**Medical Roles**	**Very Poor**	**Poor**	**Average**	**Good**	**Very Good**	**Number of Answers**
	**Personnel directly involved in patient care**	**7.6%**	**12.6%**	**31.5%**	**35.2%**	**13.1%**	**406**
	Nurses	6.3%	10.6%	31.0%	38.0%	14.2%	303
	Physicians	12.1%	22.7%	28.8%	25.8%	10.6%	66
	Physiotherapists	14.3%	-	50.0%	28.6%	7.1%	14
	Paramedics	50.0%	-	-	50.0%	-	4
	Midwives	-	9.1%	54.5%	36.4%	-	11
	Psychologists	-	66.7%	33.3%	-	-	3
	Medical caretake	-	20.0%	20.0%	40.0%	-	5
	**Personnel not directly engaged in patient care**	**1.6%**	**9.0%**	**42.6%**	**31.1%**	**15.6%**	**122**
	Administrative staff of medical institutions	2.1%	4.1%	42.3%	34.0%	17.5%	97
	Laboratory diagnosticians	-	30.0%	30.0%	30.0%	10.0%	10
	Technicians	9.1%	18.2%	45.5%	18.2%	9.1%	11
	Pharmacists	-	50.0%	50.0%	-	-	6
B. Do you think WHO recommendations should be more closely adapted to local realities?	**Medical Roles**	**Yes**	**No**	**I Don’t Know**			**Number of Answers**
	**Personnel directly involved in patient care**	**81.5%**	**6.9%**	**11.6%**			**406**
	Nurses	81.5%	6.6%	11.9%			303
	Physicians	78.5%	10.8%	10.8%			65
	Physiotherapists	92.9%	7.1%	-			14
	Paramedics	80.0%	-	20.0%			5
	Midwives	81.8%	-	18.2%			11
	Psychologists	66.7%	-	33.3%			3
	Medical caretake	100.0%	-	-			5
	**Personnel not directly engaged in patient care**	**76.4%**	**4.9%**	**18.7%**			**123**
	Administrative staff of medical institutions	76.5%	4.1%	19.4%			98
	Laboratory diagnosticians	80.0%	10.0%	10.0%			10
	Technicians	66.7%	11.1%	22.2%			9
	Pharmacists	83.3%	-	16.7%			6

**Table 6:** Selected responses of the survey, incorporating results by gender and age groups (excluding <25 years). Panel A shows the proportion of respondents who were familiar with the WHO document. Panel B presents the reported sources of information about the document. Panel C illustrates respondents’ ratings of how the WHO guidance influenced their sense of safety at work. Panel D identifies the main difficulties encountered in applying the recommendation. Panel E shows whether respondents continue to follow the 2020 WHO recommendations in current practice. Panel F shows respondents’ assessments of support received from national and international institutions in implementing personal protective equipment guidance. Panel G summarizes the main suggestions provided by respondents regarding the use of personal protective equipment in future emergencies, including epidemics, natural disasters, and other crisis situations.

A. Were you familiar with the WHO recommendations on the rational use of PPE from December 2020?	**Participant Demographics**	**Yes**	**No**	**Don’t Remember**			**Number of Answers**
	Men	64.3%	11.4%	24.3%			70
	Women	78.6%	7.1%	14.3%			462
	25–40 years	68.6%	14.4%	16.9%			118
	41–55 years	75.6%	9.1%	15.3%			242
	>55 years	83.1%	1.7%	15.3%			177
B. How did you become acquainted with the content of this document?	**Participant Demographics**	**Professional Training**	**Through the Employer**	**Independent Reading**	**I have Not Become Acquainted with It**	**Other**	**Number of Answers**
	Men	7.1%	37.1%	28.6%	18.6%	8.6%	70
	Women	16.5%	49.6%	21.2%	8.4%	4.3%	462
	25–40 years	7.6%	49.2%	17.8%	20.3%	5.1%	118
	41–55 years	12.4%	43.0%	28.1%	9.5%	7.0%	242
	>55 years	24.3%	53.1%	16.9%	4.0%	1.7%	177
C. Did applying the WHO guidelines improve your sense of safety in the workplace during the pandemic?	**Participant Demographics**	**Strongly Disagree**	**Disagree**	**Neither**	**Agree**	**Strongly Agree**	**Number of Answers**
	Men	6.0%	4.5%	40.3%	19.4%	29.9%	67
	Women	2.9%	4.9%	22.0%	34.7%	35.6%	450
	25–40 years	3.5%	3.5%	30.1%	35.4%	27.4%	113
	41–55 years	4.6%	5.9%	22.8%	33.3%	33.3%	237
	>55 years	1.7%	4.0%	22.3%	29.7%	42.3%	175
D. What were the greatest difficulties in applying WHO recommendations in your workplace during the pandemic?	**Participant Demographics**	**Staff Shortages**	**Equipment Shortages**	**Lack of Clear Local Guidelines**		**Other**	**Number of Answers**
	Men	38.5%	21.5%	33.8%		6.2%	65
	Women	51.2%	16.3%	17.9%		14.6%	459
	25–40 years	49.6%	21.4%	18.8%		10.3%	117
	41–55 years	48.5%	17.2%	23.0%		11.3%	239
	>55 years	52.3%	13.6%	15.9%		18.2%	176
E. Do you currently continue to follow the WHO recommendations from 2020 regarding PPE use?	**Participant Demographics**	**Yes**	**Partially**			**No**	**Number of Answers**
	Men	37.9%	43.9%			18.2%	66
	Women	53.9%	41.3%			4.9%	453
	25–40 years	36.8%	53.0%			10.3%	117
	41–55 years	50.6%	40.9%			8.5%	235
	>55 years	64.6%	33.7%			1.7%	175
F. How would you rate institutional support (e.g., from the Ministry of Health, WHO, Chief Sanitary Inspectorate) in implementing PPE recommendations?	**Participant Demographics**	**Very Poor**	**Poor**	**Average**	**Good**	**Very Good**	**Number of Answers**
	Men	17.6%	19.1%	26.5%	27.9%	8.8%	68
	Women	4.7%	10.5%	35.4%	35.6%	13.8%	449
	25–40 years	8.6%	16.4%	40.5%	22.4%	12.1%	116
	41–55 years	3.1%	12.9%	34.4%	36.6%	12.9%	224
	>55 years	4.0%	8.0%	31.3%	41.5%	15.3%	176
G. Do you think WHO recommendations should be more closely adapted to local realities?	**Participant Demographics**	**Yes**	**No**			**I Don’t Know**	**Number of Answers**
	Men	72.1%	13.2%			14.7%	68
	Women	82.2%	5.1%			12.7%	450
	25–40 years	79.3%	6.9%			13.8%	116
	41–55 years	82.6%	5.9%			11.4%	236
	>55 years	78.7%	6.3%			14.9%	174

## Data Availability

The datasets generated during and/or analyzed during the current study are available from the corresponding author upon reasonable request.

## Supplementary Materials

The following supporting information can be downloaded at https://www.mdpi.com/article/10.3390/jcm15030988/s1, Individual survey questions (Polish version).

## Abbreviations

The following abbreviations are used in this manuscript:

WHO	World Health Organization
PPE	Personal protective equipment
COVID-19	Coronavirus disease 2019
PSZ	Hospital Service Provision
FFP	Filtering Face Piece
