# Supplementary material for: The COVID-19 Pandemic as a Lesson: WHO Actions Versus the Expectations of Medical Staff—Evidence from Poland
Source: J Clin Med. 2026 Jan 26;15(3):988. doi: 10.3390/jcm15030988 (PMC12897928; doi:10.3390/jcm15030988)
Supplement: Supplementary file 1 [file jcm-15-00988-s001.zip › jcm-4004054-supplementary.pdf]

Supplementary 1. Polish version of individual survey questions.

Tytuł: Wykorzystanie zaleceń WHO dotyczących racjonalnego użycia Środków Ochrony Indywidulanej (ŚOI) przez personel medyczny w czasie i po pandemii COVID-19

Ankieta ma charakter **anonimowy** i **dobrowolny**, a jej wypełnienie nie powinno zająć więcej niż **10 minut**

Ankietę opracowano na podstawie dokumentu: ***World Health Organization. Rational use of personal protective equipment for covid-19 and considerations during severe shortages: Interim guidance, 23 December 2020. Geneva***. Ankieta skierowana jest do osób z personelu medycznego, które pracowały z pacjentami w okresie pandemii COVID-19 oraz po tym okresie. Ankieta jest anonimowa i dobrowolna. Jej celem jest ocena praktycznego zastosowania przez personel medyczny wytycznych WHO podczas trwania incydentów choroby zakaźnej (pandemia, epidemia) oraz wdrażania tych wytycznych w okresach poza tymi incydentami.

1. **Jaki jest Pana/i zawód medyczny?**
   - Lekarz
   - Pielęgniarka / Pielęgniarz
   - Ratownik medyczny
   - Fizjoterapeuta
   - Pracownik administracji placówki medycznej
   - Inna odpowiedź…
2. **Jak długo pracuje Pan/i w zawodzie medycznym?**
   - < 5 lat
   - 5–10 lat
   - 11–20 lat
   - >20 lat
3. **W jakiej placówce Pan/i pracuje najczęściej?**
   - Szpital
   - Przychodnia POZ
   - Specjalistyczna praktyka lekarska
   - Własna praktyka
   - Inna odpowiedź…
4. **Wiek**
   - <25 lat
   - 25–40 lat
   - 41–55 lat
   - >55 lat
5. **Płeć**
   - kobieta
   - mężczyzna
6. **Czy był/a Pan/i zaznajomiony/a z zaleceniami WHO dotyczącymi racjonalnego użycia ŚOI z grudnia 2020 r. oraz późniejszymi aktualizacjami dokumentu?**
   - Tak

- Nie
- Nie pamiętam

7. **Jak zapoznał/a się Pan/i z treścią tego dokumentu?**
    - Szkolenie zawodowe
    - Za pośrednictwem pracodawcy
    - Samodzielna lektura
    - Nie zapoznałem/am się
    - Inna odpowiedź…
8. **Czy dokument WHO był uwzględniany przy tworzeniu procedur lokalnych lub szpitalnych?**
    - Tak
    - Częściowo
    - Nie
    - Nie wiem
9. **Czy Pana/i placówka wdrożyła zalecenia WHO z powyższego dokumentu?**
    - Tak
    - Częściowo
    - Nie
    - Nie wiem
10. **Jak ocenia Pan/i dostępność ŚOI (np. masek, rękawiczek, fartuchów) w miejscu pracy podczas pandemii?**

Bardzo zła Bardzo dobra

1 2 3 4 5

11. **Których ŚOI najczęściej brakowało w Twoim miejscu pracy podczas pandemii? (można zaznaczyć kilka)**
    - Maski chirurgiczne
    - Maski FFP2/FFP3
    - Przyłbice/gogle
    - Rękawiczki
    - Fartuchy ochronne
    - Inna odpowiedź…
12. **Czy były organizowane szkolenia dotyczące racjonalnego wykorzystania ŚOI w Pana/i miejscu pracy podczas pandemii?**
    - Tak, regularnie
    - Tak, kilkukrotnie
    - Nie
    - Nie wiem
13. **Czy stosowanie wytycznych WHO poprawiło poczucie bezpieczeństwa w Pana/i miejscu pracy podczas pandemii?**

Zdecydowanie nie Zdecydowanie tak

1 2 3 4 5

14. **Jakie były największe trudności w stosowaniu zaleceń WHO w Pana/i miejscu pracy podczas pandemii?**

    - Braki kadrowe
    - Braki sprzętowe

- Brak jasnych wytycznych lokalnych
- Inna odpowiedź…

15. **Czy obecnie nadal stosuje się Pan/i do zaleceń WHO z 2020 r. i ich późniejszych aktualizacji dotyczących używania ŚOI?**

- Tak
- Częściowo
- Nie

16. **Czy uważa Pan/i, że dokument WHO z 2020 r. i jego późniejsze aktualizacje przyczyniły się do poprawy bezpieczeństwa personelu i pacjentów w aktualnej praktyce medycznej?**

Zdecydowanie nie Zdecydowanie tak

1 2 3 4 5

17. **Czy dokument WHO z 2020 r. i jego późniejsze aktualizacje wpłynęły na obecną politykę zakupów i zarządzania ŚOI w Pana/i miejscu pracy?**
    - Tak
    - Nie
    - Nie wiem
18. **Które zmiany w procedurach ŚOI z przytaczanego dokumentu WHO z 2020 r. wraz jego późniejszymi aktualizacjami powinny Pana/i zdaniem pozostać wdrożone na stałe?**
    - Racjonalne wykorzystanie ŚOI
    - Możliwość powtórnego wykorzystania ŚOI w przypadku braku tych środków w ciągu dostaw
    - Wdrażanie lokalnych zaleceń wykorzystania ŚOI
    - Wdrażanie ogólnokrajowych zaleceń wykorzystania ŚOI
    - Konieczność zapewniania ciągłości dostaw środków ŚOI
    - Inna odpowiedź…
19. **Czy uważa Pan/i, że dokument WHO z 2020 r. wraz jego późniejszymi aktualizacjami był przydatnym źródłem informacji w praktyce klinicznej?**
    - Tak
    - Częściowo
    - Nie
20. **Czy wydane w trakcie trwania pandemii wytyczne WHO zwiększyły Pana/i świadomość w zakresie racjonalnego używania ŚOI?**
    - Tak, znacznie
    - Tak, nieznacznie
    - Nie
21. **Czy uważa Pan/i, że dokument WHO z 2020 r., opisujący racjonalne wykorzystanie ŚOI pozostaje aktualny w kontekście wykonania zawodów medycznych ?**
    - Tak
    - Częściowo
    - Nie
22. **Jak ocenia Pan/i wsparcie instytucjonalne (np. Ministerstwo Zdrowia, WHO, Główny Inspektor Sanitarny) w zakresie wdrażania zaleceń dotyczących ŚOI?**

Bardzo słabe Bardzo dobre

1 2 3 4 5

23. Czy Pana/i zdaniem zalecenia WHO powinny być w większym stopniu dostosowane do realiów lokalnych?
- Tak
- Nie
- Nie wiem

24. Jakie są Pana/i ogólne wnioski lub sugestie dotyczące stosowania ŚOI w przyszłych sytuacjach kryzysowych (m.in. epidemie, klęski żywiołowe, katastrofy antropogeniczne, działania zbrojne, itp.)?
